# Improving series convergence: the simple pendulum and beyond

**DOI:** 10.1088/1361-6404/aad876

**Published:** 2018-09-11

**Authors:** Solomon F Duki, T P Doerr, Yi-Kuo Yu

**Affiliations:** National Center for Biotechnology Information, National Library of Medicine, National Institutes of Health, Bethesda, MD 20894, United States of America

**Keywords:** series convergence, numerical methods, simple pendulum

## Abstract

A simple and easy to implement method for improving the convergence of a power series is presented. We observe that the most obvious or analytically convenient point about which to make a series expansion is not always the most computationally efficient. Series convergence can be dramatically improved by choosing the center of the series expansion to be at or near the average value at which the series is to be evaluated. For illustration, we apply this method to the well-known simple pendulum and to the Mexican hat type of potential. Large performance gains are demonstrated. While the method is not always the most computationally efficient on its own, it is effective, straightforward, quite general, and can be used in combination with other methods.

## 1. Introduction

Exact solutions in physics are informative, useful, satisfying, and rare. Because relatively few problems of interest can be solved exactly, series expansions are a ubiquitous tool in physics. For example, perturbation theory is used to analyze problems that are in some sense close to a known reference problem. Series methods are used to solve the differential equations that arise in almost all areas of physics.

While a series will occassionally be exactly summable, most of the time one must proceed numerically in order to get specific results. If the series in question does not depend an any parameters and therefore sums to a single unique number, computational efficiency will usually not be an issue. However, it often happens that the series is a function of one or more physical parameters, in which case it may be necessary to compute the sum many times for various parameter values. If the series is slowly converging, computational efficiency can become an issue.

There are a number of known ways to address the problem of slow convergence. Kummer’s comparison method essentially uses a series with a known sum and with similar leading behavior to remove the slowest converging part of the series [1]. Euler’s method is a transformation to terms proportional to differences of the original terms [1]. There are further, more sophisticated transformations [2]. It is not always clear whether a given transformation method will work.

There is often a natural point about which to make a series expansion. For example, it is natural to expand $\frac{1}{1-x}$ about *x =* 0:

$$\frac{1}{1-x}=1+x+x^{2}+x^{3}+\ldots \;\;\;\;\;\text{for}|x|<1.$$

When ∣*x*∣ is close to 0 this series converges well, but the convergence gets considerably worse as ∣*x*∣ → 1. (Outside the radius of convergence of 1, say if *x* < −1, the series diverges.) Such a situation poses only minor difficulty if one knows ahead of time at which values one wishes to evaluate the series. Simply perform the expansion about a convenient nearby point.

However, in some numerical situations, it may arise that one does not know ahead of time the exact values for which one wants to sum the series or the values may be spread out in an inconvenient way. Although one could try one of the standard approaches mentioned above to speed convergence, we present here an alternative method. In any particular case, one of the methods mentioned above might (or might not) be numerically faster when used alone. However, the method we present, while still quite effective, is of broad general applicability while also possessing exceptional conceptual simplicity, making it is very easy to implement as a program. Furthermore, nothing prevents this method from being used *in addition* to other methods, thereby yielding easily obtained extra efficiency. The idea, used and illustrated in section 3.5 of [3], is to generate a series about an *average* or *representative* value that might be needed. We emphasize that an exact average value is not needed. In order to be effective, the method only requires that the point about which the expansion is made is sufficiently representative. Thus, if one wants the expectation value *E* in the example above, one may expand about some representative value ⟨*x*⟩ which need not be the exact mean of the values of *x*:

(1) $$\begin{array}{ll} E\left[\frac{1}{1-x}\right] & =E\left[\frac{1}{1-\langle x\rangle +(\langle x\rangle -x)}\right]\;\\ & =\frac{1}{1-\langle x\rangle }E\left[\frac{1}{1+(\langle x\rangle -x)/(1-\langle x\rangle )}\right]. \end{array}$$

Although this idea is quite simple and effective, it does not seem to be widely recognized and perhaps deserves wider mention as a general and straightforward numerical method. In particular, the method will be useful in upper level undergraduate courses in which series solutions become common.

## 2. Applications

To illustrate the application of our methods we use two examples. We first consider the simple pendulum [4], a well-known problem that is still the subject of considerable interest, as indicated by the various approximations, series solutions, and iterative methods that have been developed in recent years [5–14]. We will apply the method of (1) to calculate the period. As another example of the method, we then calculate the partition function for a two-dimensional system where there is a generic Mexican hat potential.

### 2.1. Simple pendulum

**Figure 1.**
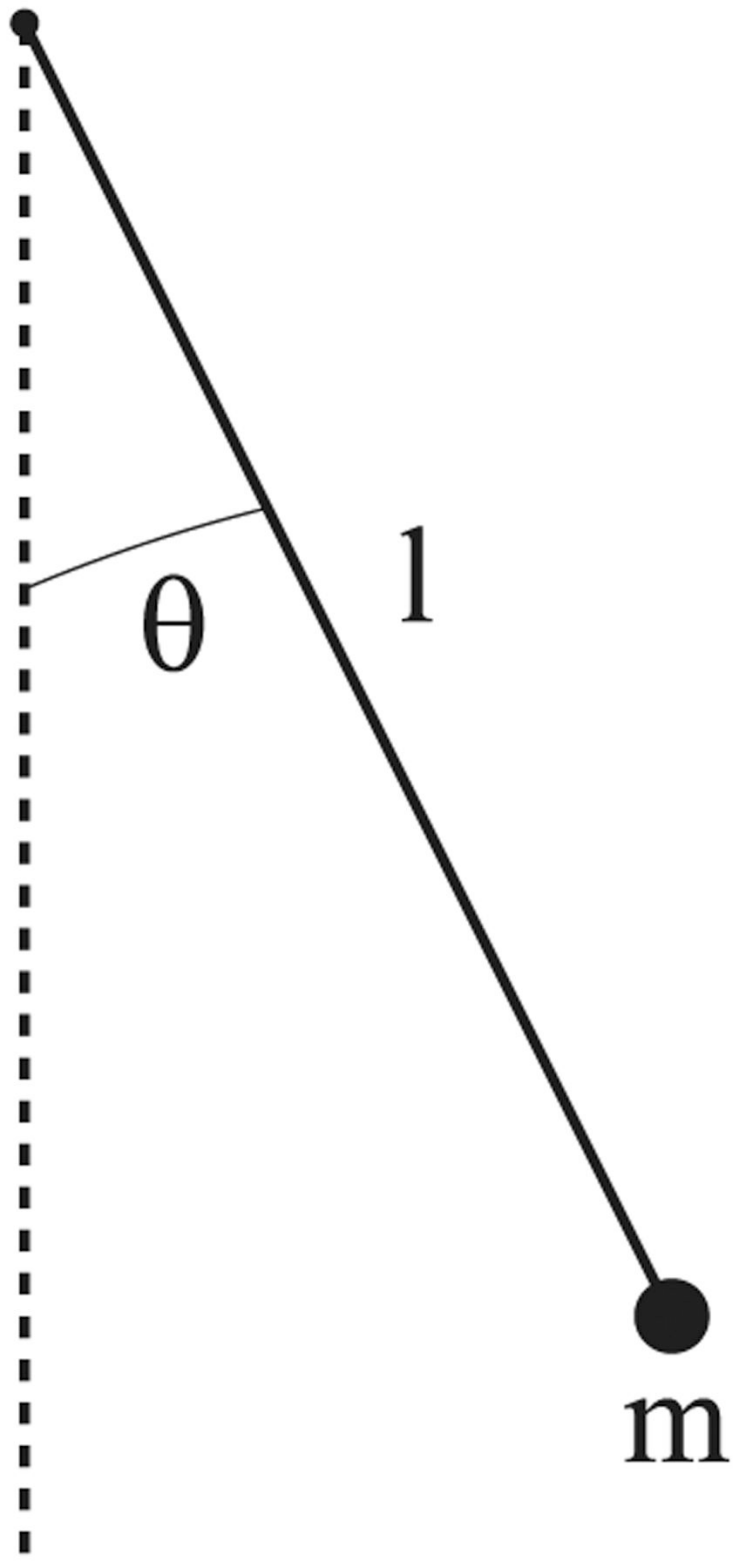
A simple pendulum. A mass *m* at the end of a massless rod of length *l* oscillates in the the plane of the page. The angle with respect to the vertical is *θ*, whose maximum value is *θ*_*m*_.

Consider a simple pendulum as shown in figure 1 with length *l* and mass *m*. Let *θ* denote the angular deviation from the vertical and let *θ*_*m*_ denote the maximum angle. For sufficiently small amplitude *θ*_*m*_, the simple pendulum becomes a simple harmonic oscillator. For larger amplitudes, however, the problem is nonlinear.

If one wishes to find the period of oscillation *T*, it is easy to start from the energy conservation equation. The potential energy at angle *θ* is *U*(*θ*) = *mgl*(1 − cos *θ*) where *g* is the acceleration of gravity and *U*(0) = 0 is the arbitrary zero level, while the kinetic energy is (1/2)*ml*^2^*θ*^2^_._ The conservation of energy equation is therefore

$$mgl\;\left(1-\text{cos}\theta_{m}\right)=\frac{1}{2}ml^{2}{\dot{\theta }}^{2}+mgl(1-\text{cos}\theta )$$

where *θ*_*m*_ is the value of *θ* at the top of each swing of the pendulum. It is easiest to try direct separation of variables for this first-order differential equation. One finds

$$\frac{\dot{\theta }}{\sqrt{\text{cos}\theta -\text{cos}\theta_{m}}}=\pm \sqrt{\frac{2g}{l}}.$$

By symmetry each quarter of a cycle is equivalent, so *T*/4 may be obtained by integrating over a quarter cycle from the *θ*(0) = 0 to *θ*(*T*/4) = *θ*_*m*_ with $\dot{\theta }\;>\;0$:

$$\int_{0}^{\theta_{m}}\frac{\text{d}\theta }{\sqrt{\text{cos}\theta -\text{cos}\theta_{m}}}=\int_{0}^{T/4}\sqrt{\frac{2g}{l}}\text{d}t.$$

The period *T* is then written in terms of an integral as

$$T=2\sqrt{\frac{2l}{g}}\int_{0}^{\theta_{m}}\frac{\text{d}\theta }{\sqrt{\text{cos}\theta -\text{cos}\theta_{m}}}.$$

With the substitution sin(*θ*/2) = sin(*θ*_*m*_/2)sin *ϕ* one may rewrite the period *T* as

$$T=4\sqrt{\frac{l}{g}}K\left({\text{sin}}^{2}\frac{\theta_{m}}{2}\right)$$

which is now in terms of the complete elliptic integral of the first kind [15],

$$K(s)\equiv \int_{0}^{\pi /2}\frac{\text{d}\phi }{\sqrt{1-s{\text{ sin}}^{2}\phi }}.$$

The elliptic integral needs to be evaluated numerically. While there are special methods for the elliptic integral [13], we are not focused here on the most effective method for the elliptic integral in particular. We are simply using the elliptic integral as an example application of a more general method for speeding series convergence.

The most obvious approach for calculating the elliptic integral is to expand the integrand as a series:

$$K(s)=\int_{0}^{\pi /2}\text{d}\phi \sum_{n=0}^{\infty }\frac{(2n-1)!!}{(2n)!!}s^{n}{\text{ sin}}^{2n}\phi$$

and integrate term by term:

(2) $$K(s)=\sum_{n=0}^{\infty }{\left(\frac{(2n-1)!!}{(2n)!!}\right)}^{2}\frac{\pi }{2}s^{n}.$$

While this expansion is good for small s, it converges very slowly for s close to 1.

If, however, one does not have any advance knowledge of *s* (other than |*s*| < 1), the strategy we propose is to expand the denominator about the presumed average value *s*/2:

$$\begin{array}{ll} K(s) & =\int_{0}^{\pi /2}\frac{\text{d}\phi }{\sqrt{1-s/2-\left(s\;{\text{sin}}^{2}\phi -s/2\right)}}\\ & =\int_{0}^{\pi /2}\frac{\text{d}\phi }{\sqrt{1-s/2}\sqrt{1-\frac{s\;{\text{sin}}^{2}\phi -s/2}{1-s/2}}}. \end{array}$$

The series expansion proceeds as before but about a more advantageous center:

$$K(s)=\frac{1}{\sqrt{1-s/2}}\sum_{n=0}^{\infty }\frac{(2n-1)!!}{(2n)!!}{\left(\frac{s}{1-s/2}\right)}^{n}\int_{0}^{\pi /2}{\left({\text{sin}}^{2}\phi -\frac{1}{2}\right)}^{n}\text{d}\phi .$$

The integral is

$$\begin{array}{ll} \int_{0}^{\pi /2}{\left(\sin^{2}\phi -\frac{1}{2}\right)}^{n}\text{d}\phi & =\frac{1}{2}{\left(-\frac{1}{2}\right)}^{n}\int_{0}^{\pi }\cos^{n}\;u\;\text{d}u\\ & =\{\begin{array}{ll} {\left(-\frac{1}{2}\right)}^{n}\frac{\pi }{2}\frac{(n-1)!!}{n!!} & n\;\text{is}\;\text{even}\\ 0 & n\;\text{is}\;\text{odd} \end{array}. \end{array}$$

The elliptic function is therefore

(3) $$K(s)=\frac{\pi }{2}\frac{1}{\sqrt{1-s/2}}\sum_{n=0}^{\infty }\frac{(4n-1)!!(2n-1)!!}{(4n)!!(2n)!!}{\left(\frac{s/2}{1-s/2}\right)}^{2n}$$

and the period is therefore

$$T=2\pi \sqrt{\frac{l}{g}}\frac{1}{\sqrt{1-s/2}}\sum_{n=0}^{\infty }\frac{(4n-1)!!(2n-1)!!}{(4n)!!(2n)!!}{\left(\frac{s/2}{1-s/2}\right)}^{2n}.$$

**Figure 2.**
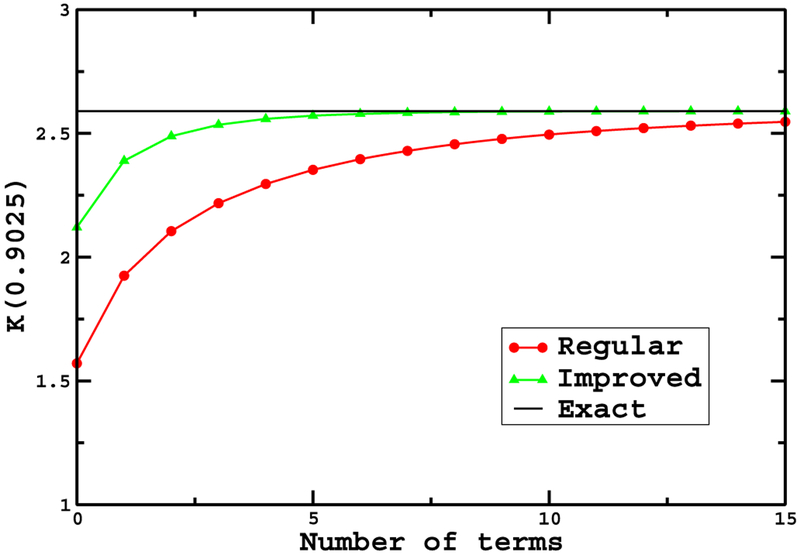
Comparison of simple expansion and improved expansion for K (0.9025). The value *s* = 0.9025 corresponds to the angle *θ* ≈ 0.798*π* radians. Points denoted by red circles correspond to the ‘regular’ expansion around *s* = 0. Points denoted by the green triangles correspond to the ‘improved’ expansion around the average value. For reference, the black horizontal line is the correct value. Roughly a quarter as many terms are needed with the improved method to obtain similar accuracy.

Figure 2 shows a comparison of *K* (0.9025) computed using the series given in (2) and the series given in (3). At the expense of only a few of lines of analysis, the convergence is much improved. The improved expansion requires only about a quarter of the number of terms in order to obtain comparable accuracy. We should note that the series in (3) is the same as in [14]. Whereas the substitution used in [14] appears inspired, we now see that it turns out to be a particular example of the more general method described here. To further demonstrate the general method of expanding about a typical value, we proceed to another example of interest.

### 2.2. Gaussian integration

As a second example we consider a two-dimensional system with a Mexican hat potential, given by

(4) $$U(r)=-ar^{2}+br^{4}.$$

Here *r*^2^=*x*^2^+*y*^2^ and *a* and *b* are positive constants. The integral we would like to evaluate is

(5) $$\Delta =\int_{0}^{\infty }e^{-\beta U(r)}d^{2}\vec{r}\;=\int_{0}^{\infty }e^{\beta ar^{2}-\beta br^{4}}rdrd\theta \;=\pi \int_{0}^{\infty }e^{\beta a\xi -\beta b\xi^{2}}d\xi \;=\pi \int_{0}^{\infty }f(\xi )e^{-\beta b\xi^{2}}d\xi$$

where ξ = *r*^2^ and *f* (ξ) = *e*^*βaξ*^. One encounters such integrals quite often in physics. For example, in the evaluation of the partition function of a ferromagnetic system, the ‘Landau free energy’ is given by a functional of the form in (4), where in this case *r* represents the order parameter [16].

In quantum field theory, this integral amounts to doing the Euclidean functional integral, where typically perturbation is done around the minimum of the Euclidean action. In two or more dimensions, the integral of this Euclidean action reveals the emergence of massless bosons due to spontaneous symmetry breaking in the scalar field (*r* in the example above) [17].

**Figure 3.**
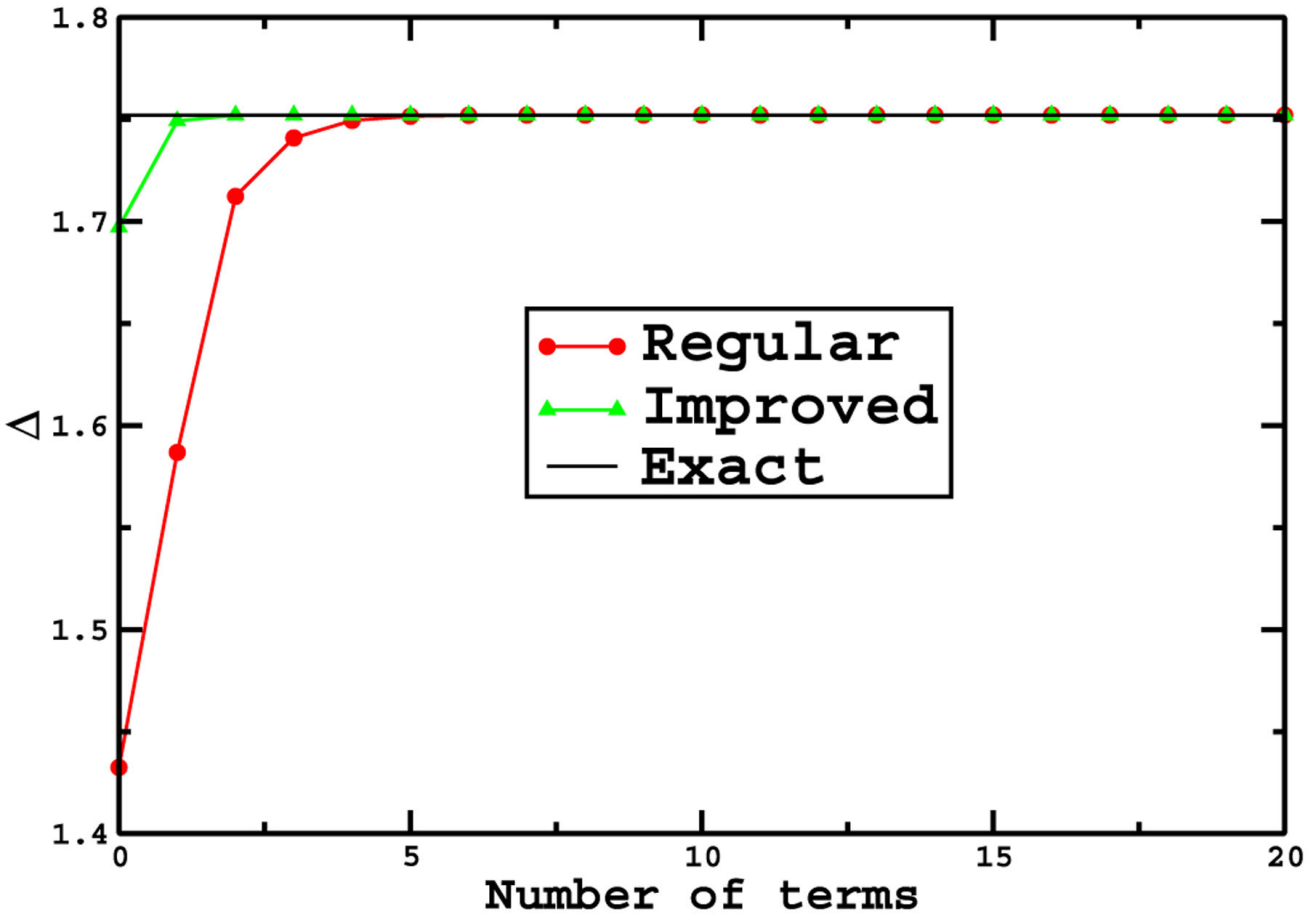
Comparison of the convergence of the integrals in equations (5) and (6) for *β* = 0.2. Points denoted by red circles correspond to the ‘regular’ expansion. Points denoted by the green triangles correspond to the ‘improved’ expansion.

**Figure 4.**
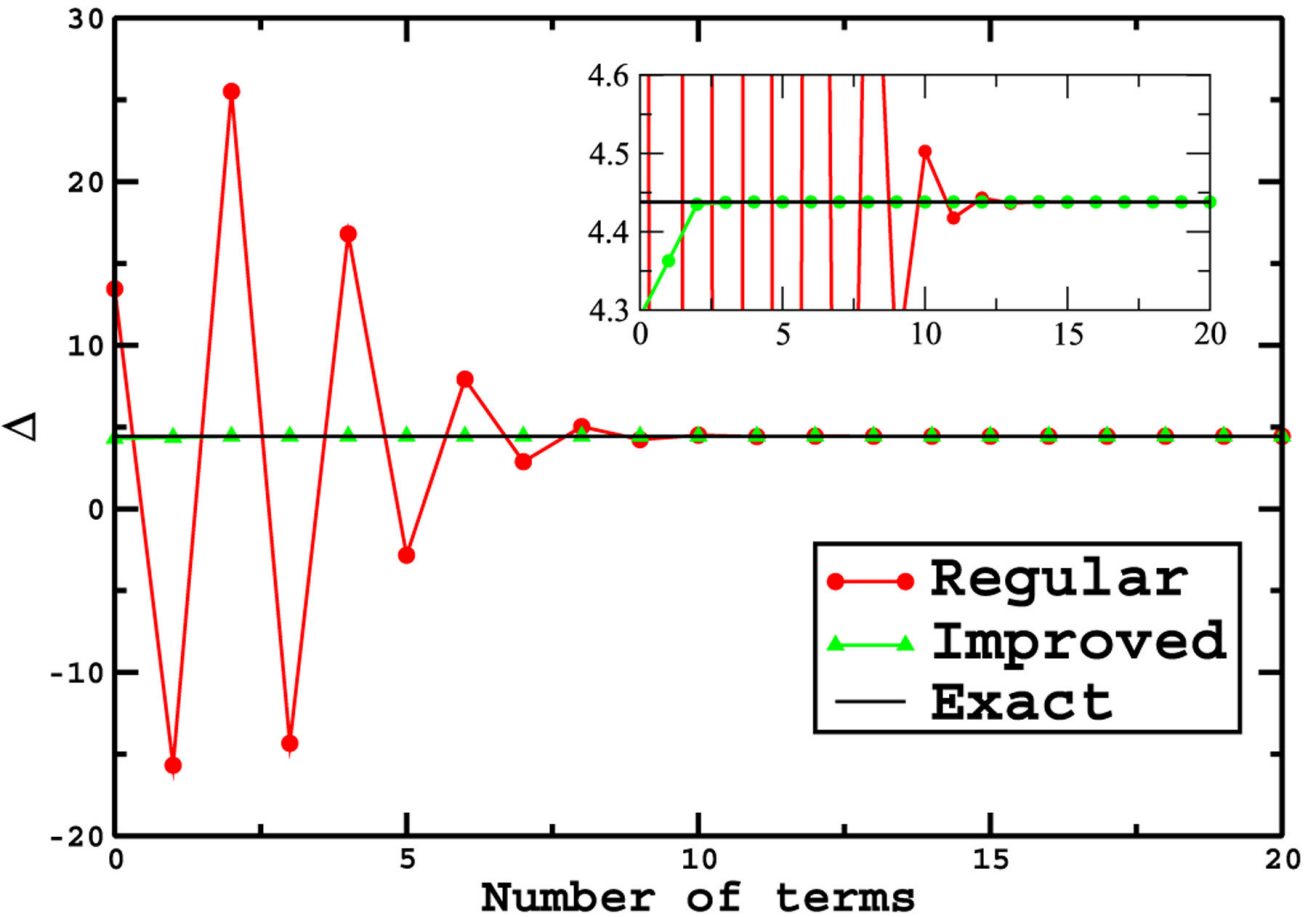
Comparison of the convergence of the integrals in equations (5) and (6) for *β* = 8. Points denoted by red circles correspond to the ‘regular’ expansion while the green triangles correspond to the ‘improved’ expansion. Magnifying the graph in a narrow range, the inset shows how wildly Δ oscillates before it converges for the ‘regular’ expansion. On the other hand the ‘improved’ method shows Δ to converge very rapidly to its exact value, 4.438066889, within the first few terms in the expansion.

The integral Δ in (5) has better convergence if one expands the potential *U*(*r*) in equation (4) around the neighborhood of some average value of $\langle r_{0}^{2}\rangle$ Hence, setting *a* = 1/2 and *b* = 1/4, equation (5) becomes

(6) $$\Delta =\frac{1}{\pi }\int_{0}^{\infty }e^{\beta \left(r^{2}-\langle r_{0}^{2}\rangle +\langle r_{0}^{2}\rangle \right)-\beta {\left(r^{2}-\langle r_{0}^{2}\rangle +\langle r_{0}^{2}\rangle \right)}^{2}}rdrd\theta \;=\gamma \int_{0}^{\infty }e^{\frac{1}{2}\beta \left(\xi -\xi_{0}\right)\left(\xi_{0}-1\right)-\frac{1}{4}\beta {\left(\xi -\xi_{0}\right)}^{2}}d\xi \;=\gamma \int_{0}^{\infty }\tilde{f}(\xi )e^{-\frac{1}{4}\beta {\left(\xi -\xi_{0}\right)}^{2}}d\xi \;=\gamma \int_{0}^{\infty }\sum_{n=0}^{m}\frac{1}{n!}{\frac{d^{n}\tilde{f}}{d\xi^{n}}|}_{\xi_{0}}{\left(\xi -\xi_{0}\right)}^{n}e^{-\frac{1}{4}\beta {\left(\xi -\xi_{0}\right)}^{2}}d\xi$$

where $\gamma =e^{\frac{1}{4}\beta \xi_{0}\left(\xi_{0}-2\right)}$ and $\tilde{f}(\xi )=e^{\frac{1}{2}\beta \left(\xi -\xi_{0}\right)\left(\xi_{0}-1\right)}$. This integration converges very rapidly by considering only a small number of terms from the sum. Figures 3 and 4 show a comparison of the convergence of the integrals (5) and (6) for *β* = 0.2 and *β* = 8 respectively. Note that the performance improvement is even better than for the simple pendulum, the results being nearly exact for just a couple of terms. Upon reflection, we expect the performance gain to improve as the dimensionality of the problem increases since a performance gain is obtained for each dimension.

## 3. Discussion

We have presented a little-mentioned but simple and easily implemented method for speeding the convergence of a series. While not necessarily the most efficient method in any particular case, the method is applicable to a wide variety of problems. We have shown that generating a series about the average value required is a simple but effective strategy. As a practical matter, one need not use the precise mean; indeed, depending on the distribution of points for which the value of the series is required, some other expansion center might be preferable. The point is simply to perform the expansion about a center that, while not necessarily the most natural, is relatively close to most of the points of interest. Any reasonable heuristic for choosing the center of expansion will do. If some center other than the mean turns out to be convenient, then by all means it should be used.

Despite its simplicity and broad applicability, the method does not seem to be commonly discussed in mathematical methods or numerical methods books. However, it is nice to have a much simpler and more intuitive method, quite elementary by comparison to the transformation methods, for example, that is also of general applicability and extremely easy to implement.
